# *Phytophthora infestans* RXLR effector *Pi04089* perturbs diverse defense-related genes to suppress host immunity

**DOI:** 10.1186/s12870-021-03364-0

**Published:** 2021-12-09

**Authors:** Ming Luo, Xinyuan Sun, Yetong Qi, Jing Zhou, Xintong Wu, Zhendong Tian

**Affiliations:** 1 Key Laboratory of Horticultural Plant Biology (HZAU), Ministry of Education, Wuhan, 430070 Hubei China; 2 Key Laboratory of Potato Biology and Biotechnology (HZAU), Ministry of Agriculture and Rural Affairs, Wuhan, 430070 Hubei China; 3 Potato Engineering and Technology Research Center (HZAU), Wuhan, 430070 Hubei China; 4 Hubei Hongshan laboratory. Huazhong Agricultural University (HZAU), No.1, Shizishan Street, Hongshan District, Wuhan, 430070 Hubei China

**Keywords:** Comparative transcriptomics, Potato, Late blight, Effector, Plant defense, PTI response

## Abstract

**Background:**

The oomycete pathogen secretes many effectors into host cells to manipulate host defenses. For the majority of effectors, the mechanisms related to how they alter the expression of host genes and reprogram defenses are not well understood. In order to investigate the molecular mechanisms governing the influence that the *Phytophthora infestans* RXLR effector *Pi04089* has on host immunity, a comparative transcriptome analysis was conducted on *Pi04089* stable transgenic and wild-type potato plants.

**Results:**

Potato plants stably expressing *Pi04089* were more susceptible to *P. infestans*. RNA-seq analysis revealed that 658 upregulated genes and 722 downregulated genes were characterized in *Pi04089* transgenic lines. A large number of genes involved in the biological process, including many defense-related genes and certain genes that respond to salicylic acid, were suppressed. Moreover, the comparative transcriptome analysis revealed that *Pi04089* significantly inhibited the expression of many flg22 (a microbe-associated molecular pattern, PAMP)-inducible genes, including various Avr9/Cf-9 rapidly elicited (ACRE) genes. Four selected differentially expressed genes (*StWAT1*, *StCEVI57*, *StKTI1,* and *StP450)* were confirmed to be involved in host resistance against *P. infestans* when they were transiently expressed in *Nicotiana benthamiana.*

**Conclusion:**

The *P. infestans* effector Pi04089 was shown to suppress the expression of many resistance-related genes in potato plants. Moreover, *Pi04089* was found to significantly suppress flg22-triggered defense signaling in potato plants. This research provides new insights into how an oomycete effector perturbs host immune responses at the transcriptome level.

**Supplementary Information:**

The online version contains supplementary material available at 10.1186/s12870-021-03364-0.

## Background

Plant–pathogen interaction consists of pathogen invasion, plant defense, and pathogen counterattack through the secretion of effectors into host cells [1]. The ‘zigzag’ model of plant immunity was proposed to describe plantpathogen interaction [2]. Conserved molecules shared by many classes of microbe are recognized by transmembrane pattern recognition receptors (PRRs), which are known as microbial- or pathogen-associated molecular patterns (MAMPs or PAMPs), and include flagellin. These cause PAMP-triggered immunity (PTI). The second layer of plant immunity is the recognition of effectors by resistance proteins (R proteins) with nucleotide-binding (NB) and leucine-rich repeat (LRR) domains. Pathogens secrete effectors into host cells to promote virulence and propagation [2]. Thereafter, cytoplasmic R proteins in the host sense the effectors, which results in hypersensitive cell death (HR) and effector-triggered immunity (ETI). Certain effectors can also be recognized in the apoplast [3].

Late blight disease is caused by the destructive oomycete pathogen *Phytophthora infestans*, which was the cause of the Irish famine. It results in damage to potato production (*Solanum tuberosum*. L) [4, 5], with severe outbreaks also threating tomato production (*Solanum lycopersicum*). During the co-evolution of the plant and pathogen, the pathogen evolved a “toolkit” with which to facilitate the manipulation of the host plant to the advantage of the microbe. The effector proteins are vital virulence determinants during pathogen infection and colonization. *P. infestans* contains more than 450 candidate RXLR effector genes [6], and shares the conserved Arg-any amino acid-Leu-Arg motif between individuals to facilitate their delivery into plant cells [7, 8]. As we currently understand it, the majority of *P. infestans* avirulence proteins, including AVR1, AVR2, AVR3a, AVR3b, AVR4, AVR8, AVRblb1, AVRblb2, AVRamr1, Avrchc1.1, and Avrchc1.2, are RXLR effectors, which are recognized by the corresponding R proteins and trigger strong immune responses [9–15]. Oomycete RXLR effectors manipulate a range of host processes by directly interacting with a diverse array of plant proteins to facilitate infection and colonization [16–18]. Certain *P. infestans* RXLR effectors target host positive regulator proteins involved in immunity. For example, the RXLR effectors PexRD2 and Pi22926 target kinase MAP 3Kɛ and MAP 3Kβ, which mediate signal transduction following perception of the *Cladosporium fulvum* effector Avr4 by the tomato Cf4 resistance protein [19, 20]. AVR3a targets the CMPG1 protein: a host ubiquitin E3 ligase that is required for cell death and is triggered by the PAMP INF1 [21, 22]. Pi03192 targets NAC transcription factors to prevent their relocalization from the endoplasmic reticulum to the nucleus to suppress immunity [23]. SFI3 targets potato UBK to suppress early PAMP-triggered immune responses [16]. Moreover, other effectors, including Pi04314 (targets phosphatase PP1C), Pi02860 (targets StNRL1), Pi17316 (targets MAP 3 K StVIK), Pi04089 (targets StKRBP1), PITG20303, and PITG20300 (targets StMKK1), interact with different negative regulators to facilitate infection and colonization [24–28]. When effectors are secreted into host cells, single effectors always target several host proteins. As regards effector function research, most studies are focused on a single effector–target interaction or a few effector–target interactions. How the *P. infestans* effector significantly perturbs the host transcriptome and thus manipulates plant immunity remains unknown. AVR2 interacts with potato BSL1: a putative phosphatase involved in brassinosteroid (BR) hormone signaling. *AVR2* transgenic potato plants exhibit transcriptional and phenotypic hallmarks of overactive BR signaling and show increased susceptibility to *P. infestans* [29]. Recently, it was reported that 190 differentially expressed genes (DEGs) were identified in *PITG15718.2* transgenic potato lines, including 158 upregulated genes and 32 downregulated genes. The downregulated DEGs in *PITG15718.2* transgenic potato may positively regulate immunity and plant growth, while the up-regulated genes may negatively regulate plant immunity or vegetative growth by decreasing the Indole-3-Acetic acid content [30].

In order to find novel strategies to control late blight disease, it is imperative to understand the potato’s responses to the *P. infestans* effector and how this pathogen manipulates host immunity. Previous studies demonstrated that the transient expression of the RXLR effector *Pi04089* in *Nicotiana benthamiana* promotes *P. infestans* colonization [27]. Pi04089 interacts with a K-homology (KH) RNA-binding protein (StKRBP1), which is a susceptibility factor. Transient expression of *StKRBP1* significantly promotes *P. infestans* colonization in *N. benthamiana* [27]. In this study, the *P. infestans* RXLR effector *Pi04089* and stable transgenic potato plants were used to investigate the transcriptional changes that occur in the potato and further explore the mechanism involved in how Pi04089 manipulates host immunity. A comparative transcriptome analysis was conducted to analyze the transcriptome difference between *Pi04089* transgenic lines and wild-type potato plants under normal conditions and flg22 treatment. Four selected genes, up- or downregulated by Pi04089, were confirmed to be involved in late blight resistance by bioassay.

## Materials and methods

### Plant material and RNA preparation

Potato (*Solanum tuberosum*) variety ‘E-potato-3’ (E3) and *Pi04089* transgenic lines were used in this work. Tubers were kindly provided by the Key Laboratory of Potato Biology and Biotechnology (HZAU), the Ministry of Agriculture and Rural Affairs. Plants were grown under standard conditions (22 to 26 °C; 16 h light/8 h dark photoperiod; and 70% relative humidity) in a greenhouse for approximately 45 days. Flg22 (QRLSSGLRINSAKDDAAGLAIS, synthesized by Sangon Biotech (Shanghai) Co., Ltd.) powder was dissolved with DMSO and diluted to 40 μmol/L with water. Detached leaves from the transgenic lines and E3 lines were sprayed with flg22/DMSO solvent for 30 min, as described in a previous work [31]. Three biological replicates were prepared. Total RNA was extracted from individual samples with the RNeasy PlantMini Kit (Qiagen) following the manufacturer’s instructions. RNA was quantified with Nanodrop (Thermo Scientific, Sugarland, TX, USA). The *N. benthamiana* seeds used in this work were preserved in the Key Laboratory of Potato Biology and Biotechnology. *N. benthamiana* were grown under standard conditions (22 °C; 16 h light/8 h dark photoperiod; and 60% relative humidity) in a chamber for approximately 3 weeks for transient expression and *P. infestans* inoculation.

### cDNA library construction and Illumina sequencing

Sequencing libraries were generated using NEBNext® UltraTM RNA Library Prep Kit for Illumina® (NEB, USA) following the manufacturer’s recommendations. mRNA was purified from total RNA using poly-T oligo-attached magnetic beads. Fragmentation was carried out using divalent cations at an elevated temperature in NEBNext First Strand Synthesis Reaction Buffer (5 X). The library fragments were purified with AMPure XPsystem (Beckman Coulter, Beverly, USA). Thereafter, PCR was performed with Phusion High-Fidelity DNApolymerase, Universal PCR primers, and Index (X) Primer. Finally, the products were purified (AMPure XP system) and library quality was assessed using the Agilent Bioanalyzer 2100 system. Clustering of index-coded samples was performed on a cBot Cluster Generation System using the Hiseq 4000 PE Cluster Kit (Illumina), according to the manufacturer’s instructions. After cluster generation, the library preparations were sequenced on an Illumina Hiseq 4000 platform and 150 bp paired-end reads were generated.

### qRT-PCR validation of candidate gene expression

Gene expression was validated with qRT-PCR. Then, 2 μg of total RNA was added to the 20 μl reaction for inverse transcription. An abm Master Mix 5 X was used to synthesize cDNA from RNA. Then, an abm EvaGreen Express 2 X qPCR MasterMix-No Dye was used to perform quantitative real-time PCR with the QuantStudio 12 K Flex Real-Time PCR system. A total of 10 μl of the mixed liquid was used for qRT-PCR (5 μl EvaGreen Express 2 X qPCR MasterMix, 1 μl cDNA, 1 μl primer and 3 μl H_2_O). The annealing temperature was set at 60 °C and a total of 40 amplification cycles with three replicates were performed for each sample. The expression level of each gene was calculated using the 2^−ΔΔCt^ method with *StEF-1α* as an internal reference gene [32]. Primers (Table S8) were designed using NCBI primer designing tools.

### Differentially expressed gene (DEGs) analysis and enrichment analysis

Paired-end clean reads were checked for quality with FastQC and then adapter trimmed with Trimmomatics [33], before mapping to the potato genome (*S. tuberosum* Group *Phureja* DM1–3516 R44) [34] using TopHat2 with a 1% mismatch [35]. The Cufflinks software tool was used to reconstruct the transcripts [36]. The calculations and differently expression analysis (FDR < 0.05 and the absolute log_2_ (fold change) > 1) were performed with cuffdiff. DEGs in this study represents differently expressed transcripts instead of genes. The gene ontology (GO) enrichment analysis was performed using the OmicShare tools: a free online platform for data analysis (http://www.omicshare.com/tools). Alternative splicing events were analyzed with rMATS [37]. Events with FDR < 0.05 were regarded as the differential AS events.

### The construction of vectors for transient expression

To clone the full-length CDS of *StKTI1*, *StWAT1*, *StCEVI57,* and *StP450* from potato E3 cDNA, four pairs of specific primers with recombinant adaptors (Table S8) were designed according to sequences deposited in NCBI. A full-length CDS with adaptors was recombined into a pH7Lic-N-GFP *Stu* I restriction site using SE recombinase and the correction of constructs was confirmed by sequencing.

### *Agrobacterium*-mediated transient expression

Constructs used in this work were transformed into the *Agrobacterium* strain GV3101 by electroporation. Bacteria picked from fresh plates were inoculated in liquid YEB and incubated overnight at 28 °C while being shaken. Agrobacterial cultures were centrifuged at 3000 rpm for 10 min, and the bacterial pellet was resuspended in 10 mM MMA 10 mM MgCl_2_ buffer. OD_600_ was adjusted to 0.1 for Agroinfiltration assays, with acetosyringone being added at 200 mM. *N. benthamiana* leaves were infiltrated using a 1-mL syringe after wounding with a needle.

### *P. infestans* inoculation

Two *P. infestans* isolates were used for inoculation. Isolate HB09–14-2 was used to infect potato leaves and isolate 88,069 was used to infect *N. benthamiana* leaves. *P. infestans* was grown on a rye agar Petri dish. To harvest the sporangia, 15-day-old plates were flooded with 5 mL of sterile distilled water and scraped with a spreader. The suspension containing sporangia was filtered through a 300/1200 mesh gauze and adjusted to 100 sporangia/μl. Thereafter, 10 μL droplets were pipetted onto the surface of the detached leaves, which were maintained in a sealed boxed with moist tissue paper. Boxes were kept in darkness for the first 24 h before being transferred to normal light conditions. Lesion diameters were measured at 6 d post-inoculation. When used in combination, *P. infestans* was inoculated 24 h after infiltration with *Agrobacterium* suspension to transiently express the selected genes.

### Statistical analysis

One-way ANOVA was used to analyze the significance. The lesion-size calculation is represented by the mean ± SD from three independent experiments. The qRT-PCR results are shown as the mean ± SEM from three biological repetitions.

## Results

### Stable expression of *Pi04089* in potato enhances *P. infestans* colonization

**Fig. 1:**
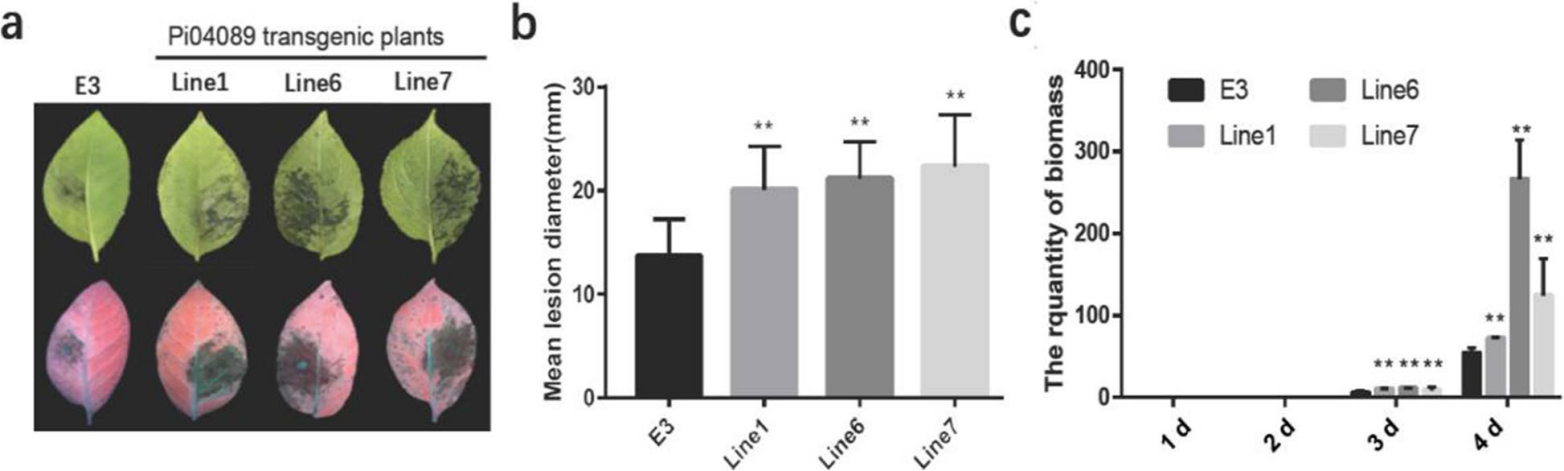
Stable expression of *Pi04089* in potato enhances susceptibility to *P. infestans*. (**a**) Representative images of leaves of the control (E3) and Pi04089-expressing lines at 5 days post-inoculation (dpi) with *P. infestans* isolate HB09–14-2. Images were photographed under normal and UV light. (**b**) Graph showing that mean lesion diameter was significantly enhanced in three transgenic potato lines as compared to the control at 5 dpi. The disease lesion diameter was measured from at least 20 leaves. ** indicates extremely significant (one-way ANOVA, *p* < 0.01) differences. Error bars indicate SD. (**c**) Dynamic hypha biomass was measured by q-PCR. Total DNA was extracted from leaves around inoculated sites after 1, 2, 3, and 4 days. The relative hypha biomass was quantified by normalizing the *P. infestans PiO8* gene values with the potato *StEF1α* gene values. ** indicates extremely significant differences (one-way ANOVA, *p* < 0.01). Error bars indicate SEM. All experiments are the combination of at least three biological replicates

Transient expression of the *P. infestans* RxLR effector *Pi04089* in *N. benthamiana* leaves significantly promoted *P. infestans* colonization [27]. To investigate whether *Pi04089* exerted the same effects on potato plants, serval transgenic potato lines were produced to stably express *Pi04089* by agrobacterial- mediated transformation. *Pi04089* was promoted by the cauliflower mosaic virus (CaMV) 35S promoter. Three lines (lines 1, 6, and 7) that demonstrated stable expression of *Pi04089* were selected for the *P. infestans* inoculation assay (Fig. S1). The *P. infestans* strain HB09–14-2 was inoculated on the detached leaves from three transgenic lines and E3 control plants. Larger disease lesions were observed on the leaves of *Pi04089* transgenic plants as compared to wild-type plants at 5 days post-inoculation (dpi) (Fig. 1a and b). In addition, the hypha biomass on transgenic plant leaves was significantly increased as compared to that of the control leaves at 4 dpi (Fig. 1c), reflecting the faster growth of the hypha on the transgenic plant leaves. These results demonstrate that *Pi04089* promotes pathogen colonization and growth in potato plants.

### Illumina sequencing and reads assembly

The stable expression of *Pi04089* in potato significantly promoted *P. infestans* growth on potato leaves, which motivated us to investigate the gene profiles in transgenic potato plants. *Pi04089* transgenic line 6 was used for further research. With the aim of investigating gene expression profile changes under normal growth conditions and responses to PAMP, leaves from transgenic plant and E3 lines under normal growth conditions and flg22 treatment were collected for RNA extraction and RNA-seq. In total, approximately 197 million paired-end reads were obtained from 12 RNA samples (three biological replicates). After quality assessment and filtering, approximately 80% of reads were mapped to the doubled monoploid potato *S. tuberosum* DM1–3 reference genome (http://solanaceae.plantbiology.msu.edu/pgsc_download.shtml) using tophat2 software. Thereafter, transcripts were reconstructed using the Cufflinks software tool, and approximately 23,000 of 38,982 genes were expressed in a total of 12 samples (Table S1); The expressions of all transcripts are shown in Table S1. Twelve up- or downregulated genes were randomly selected in order to verify their expression using qRT-PCR. The results show that the qRT-PCR qualification is consistent with the RNA-seq data (Fig. S2). In addition, many novel transcripts were found in the RNA seq data.

### Transcriptional profile changes in *Pi04089* transgenic potato plants

To explore the global effects that the effector *Pi04089* has on host plants, gene expression profiles were analyzed in *Pi04089* transgenic potato plants and compared with the E3 control plants under normal conditions. The transcriptome comparison analysis revealed that 1380 genes were identified as being differentially expressed genes (DEGs), among which 658 transcripts were upregulated and 722 transcripts were downregulated in the *Pi04089* transgenic plants (Table S2).

**Fig. 2:**
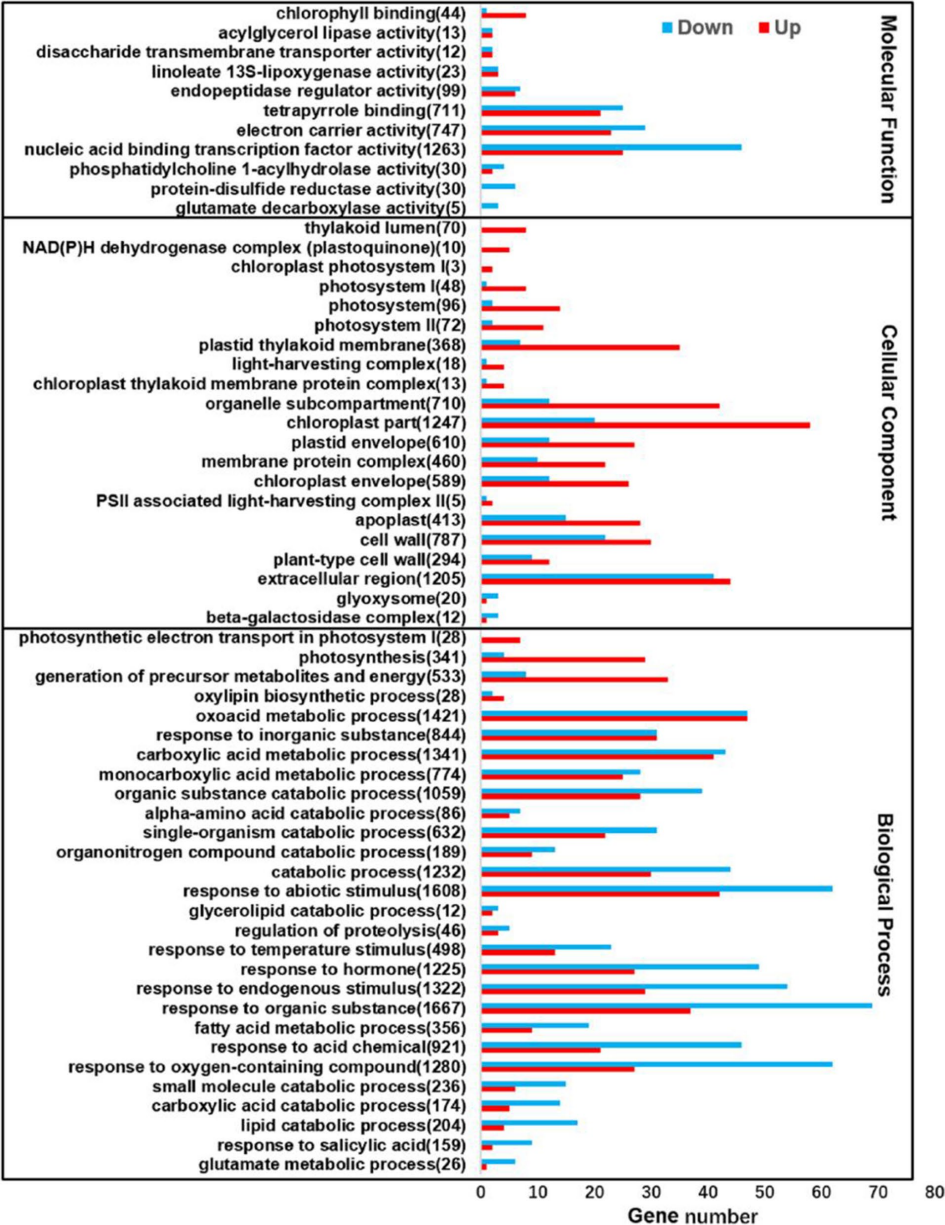
GO analysis of DEGs in *Pi04089* transgenic potato plants (generated with omicshare). The bar indicates the number of genes within each GO term, and the red and blue colored bars indicate the upregulated and downregulated genes. The number in brackets indicates the background gene number in the GO term. The adjusted *p*-values for GO terms < 0.05. See Supplementary Table 3 for the full GO terms

To understand the global biological functions of the DEGs trigged by *Pi04089*, GO analysis was performed to display the transcriptome changes (Fig. 2). The results show that the upregulated genes induced by *Pi04089* mainly involved an in cellular component, such as the photosystem, plastid thylakoid membrane, NAD(P) H dehydrogenase complex (plastoquinone), light-harvesting complex, or membrane protein complex. The majority are related to photosystem, which is perhaps caused by changes in plant physiology and growth. Moreover, the genes suppressed by *Pi04089* are involved in biological process and molecular function, such as genes related to protein-disulfide reductase activity, glutamate decarboxylase activity, nucleic acid binding transcription factor activity, the acylglycerol catabolic process, and the glutamate metabolic process. In particular, gene responses to abiotic stimulus, salicylic acid, hormone, and oxygen-containing compounds, and genes involved in the lipid catabolic process and small molecule metabolic process were significantly downregulated in *Pi04089* transgenic plants. The majority are related to plant immunity against pathogens and abiotic stress.

**Fig. 3:**
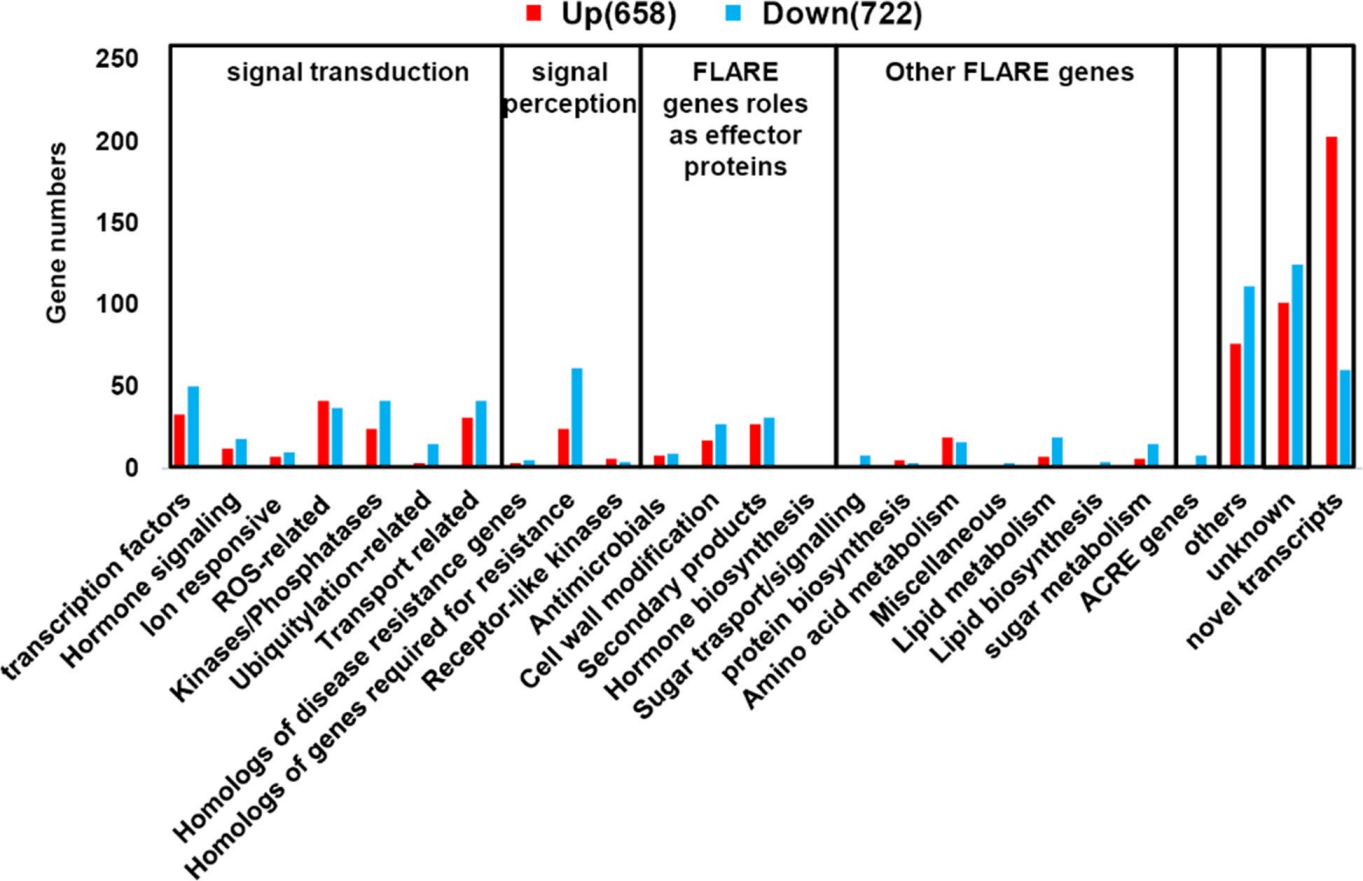
Classification of DEGs triggered by *Pi04089*. DEGs were classified into eight classes according to the annotation. FLARE: flg22 rapidly elicit genes. ACRE: Avr9/Cf-9 rapidly elicited genes. Unknown represents genes without annotation. Novel transcripts represent genes mapped into intergenic regions

In order to provide a detailed view of these differently expressed genes, all DEGs were divided into different classes (Table S2). From the 662 downregulated genes, there are many resistance-related genes involved in signal perception and signal transduction. ACRE (Avr9/Cf-9 rapidly elicited) genes and FLARE (flg22 rapidly elicited) genes have been shown to be involved in plant immunity [31]. We found that eight ACRE genes and many signal perception genes were downregulated in *Pi04089* transgenic plants (Fig. 3, Table S2). In addition, genes required for resistance, ubiquitination-related genes, and lipid metabolism genes were downregulated. Many studies demonstrate the importance of TFs in the regulation of plant immunity [38]. Fifty transcripts coding various transcription factors, such as WRKY, AP2/ERF, NAC, and Zinc finger protein, were suppressed in *Pi04089* transgenic potato plants. Genes involved in hormone signaling, kinases/phosphatases, transport, and ion responses were also significantly downregulated in *Pi04089* transgenic lines (Table S2). Moreover, approximately 200 novel transcripts were also upregulated. The Above results demonstrate that the expression of *Pi04089* in potato plants profoundly suppresses host defense-response genes.

**Fig. 4:**
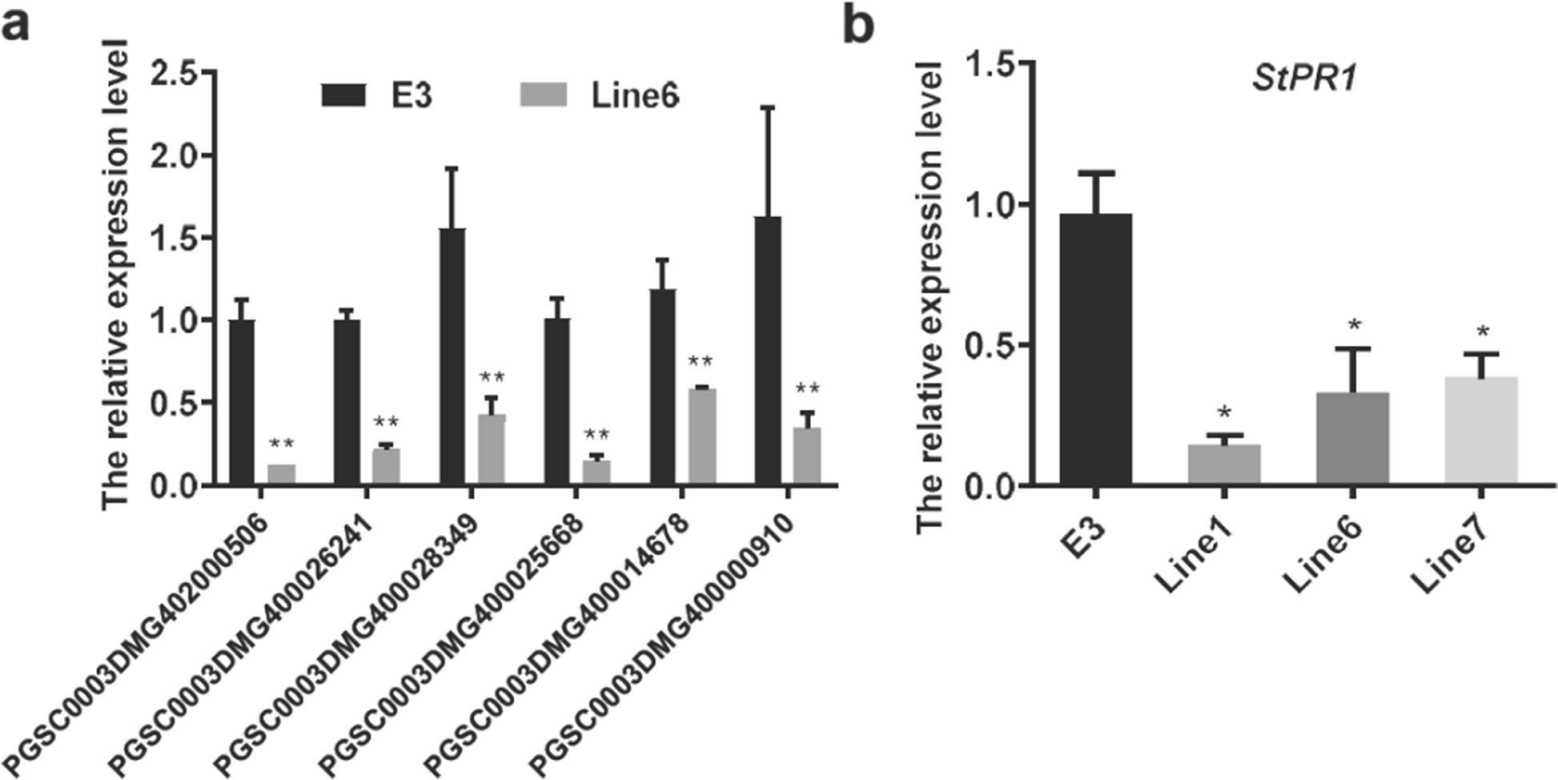
Relative expression of gene responses to salicylic acid in the *Pi04089* transgenic potato line and the E3 control line. (**a**) Expression of six genes enriched in the pathway that responds to salicylic acid in *Pi04089* transgenic plants. Gene-specific primers were designed to quantity the expression of six genes. The expression level was normalized to *StEF* and is shown relative to the control plant. Six genes were significantly downregulated in *Pi04089* transgenic plants as compared to control plants (one-way ANOVA, **, *p* < 0.01). (**b**) The *PR1* gene (PGSC0003DMG400005111) was downregulated in three transgenic lines (one-way ANOVA, *, *p* < 0.05). The experiment was conducted using three biological repeats

We noted that nine gene responses to salicylic acid (GO: 0009751) were downregulated in *Pi04089* stable transgenic plants (Table S3). Among them, six genes, i.e., coding for AP2/ERF domain-containing transcription factor (PGSC0003DMG400000910), Alpha-DOX2 (PGSC0003DMG402000506), MybSt1 (PGSC0003DMG400026241), ATP binding protein (PGSC0003DMG400025668), Flavonol 4′-sulfotransferase (PGSC0003DMG400028349), and Serine/threonine-protein kinase cx32 (PGSC0003DMG400014678), all of which were supposed to be enriched in the pathway response to salicylic acid, were confirmed to be significantly downregulated in *Pi04089* stable transgenic line 6 by qRT-PCR (Fig. 4a). The *PR1* gene is a well-known marker gene involved in the salicylic acid signal pathway. *PR1* (PGSC0003DMG400005111) was significantly downregulated in three *Pi04089* transgenic lines (Fig. 4b). In summary, the results indicate that *Pi04089* might suppress plant immunity by inhibiting the salicylic acid response pathway.

### flg22 triggers the expression of diverse defense-related genes in potato plants

As the above results show, many genes involved in PTI, including ACRE and FLARE genes, were downregulated in *Pi04089* transgenic potato plants under normal conditions (Fig. 3, Table S2). Thereafter, we explored whether *Pi04089* altered the responsive ability of potato to PAMPs. Flg22 was used to treat *Pi04089* transgenic and control E3 potato leaves. The differentially expressed genes induced by flg22 were compared between the *Pi04089* transgenic plants and the E3 controls.

First, we analyzed the gene expression profile of the E3 control potato line in response to flg22. In total, 685 upregulated transcripts and 122 downregulated transcripts were found in the E3 line (Table S4). As regards the 685 upregulated genes, the majority belong to transcription factors, secondary products related to FLARE gene roles, resistant genes, hormone signaling, ion responses, and ROS. Interestingly, the fact that 19 ACRE genes were significantly upregulated indicates that the genes that responded to Avr9/Cf9 were also triggered by flg22. Various other defense-related genes were also highly expressed upon flg22 induction, such as chitinase, Alpha-DOX2, and ATP binding protein gene. The expression levels of eight flg22-response genes were confirmed by qRT-PCR (Fig. S3).

To further investigate how flg22 triggers potato immunity, GO enrichment analysis was performed on the flg22-induced DEGs in the E3 line (Table S3). In terms of biological processes, gene responses to endogenous stimuli (GO: 0009719), to oxygen-containing compound (GO: 1901700), to chitin (GO: 0010200), to organonitrogen compound (GO: 0010243), to ethylene (GO: 0009723), to jasmonic acid (GO: 0009753) and to salicylic acid (GO: 0009751) were upregulated (Table 1). As regards molecular function, genes involved in calcium ion binding (GO: 0005509) and carboxy-lyase activity (GO: 0016831) were upregulated. Moreover, regarding cellular components, genes related with the cell periphery (GO: 0071944), plasma membrane (GO: 0005886), cell wall (GO: 0005618), and the external encapsulating structure (GO: 0030312) were downregulated. It is obvious that, as a PAMP, flg22 triggers a stronger defense response in potato plants, as is demonstrated by the amount of upregulated DEGs, which include many well-known PTI genes, genes that respond to biotic/abiotic stress, and important genes in secondary metabolite synthesis/metabolism.

**Table 1:** GO analysis of DEGs triggered by flg22 in the E3 line

	class	ID	Description	bg_num	Qvalue	fg_num
**Up regulated**	Biological Process	GO:0010200	response to chitin	122	2.97E-21	30
		GO:0010243	response to organonitrogen compound	153	1.33E-19	31
		GO:1901698	response to nitrogen compound	263	1.78E-14	33
		GO:1901700	response to oxygen-containing compound	1280	3.71E-09	64
		GO:0009719	response to endogenous stimulus	1322	1.61E-06	59
		GO:0006558	L-phenylalanine metabolic process	20	1.10E-05	7
		GO:0009800	cinnamic acid biosynthetic process	9	4.18E-05	5
		GO:0009723	response to ethylene	261	1.07E-02	15
		GO:0009753	response to jasmonic acid	180	1.09E-02	12
		GO:0009751	response to salicylic acid	159	1.48E-03	13
		GO:0006571	tyrosine biosynthetic process	9	1.37E-03	4
	Molecular Function	GO:0045548	phenylalanine ammonia-lyase activity	9	1.33E-04	5
		GO:0004398	histidine decarboxylase activity	12	2.40E-04	5
		GO:0016831	carboxy-lyase activity	95	2.40E-04	11
		GO:0005509	calcium ion binding	284	2.60E-04	19
**Down regulated**	Cellular Component	GO:0071944	cell periphery	3388	2.30E-02	29
		GO:0046658	anchored component of plasma membrane	80	2.30E-02	4
		GO:0031225	anchored component of membrane	168	3.24E-02	5
		GO:0005886	plasma membrane	2741	4.26E-02	23
		GO:0031226	intrinsic component of plasma membrane	126	4.26E-02	4
		GO:0005618	cell wall	787	4.26E-02	10
		GO:0030312	external encapsulating structure	788	4.26E-02	10

**bg_num: background gene number; fg_num: enriched gene number

### *Pi04089* inhibits the expression of defense-related genes responding to flg22

In flg22-treated *Pi04089* stable transgenic potato plants, 495 transcripts were upregulated and 96 transcripts were downregulated (Table S5). When those DEGs were compared with those in the flg22- treated control potato E3 line, 312 transcripts were upregulated and 30 transcripts were downregulated in both the *Pi04089* stable transgenic and E3 control potato plants. These overlapping genes may reflect the common responses of *Pi04089* stable transgenic and control potato plants to the PAMP flg22. Moreover, 369 genes were upregulated and 91 genes were downregulated solely in the E3 control line. In addition, 181 transcripts were upregulated and 62 transcripts were downregulated solely in the *Pi04089* transgenic line (Fig. S4).

GO analysis was performed to investigate the pathway-enriched common DEGs in flg22-treated transgenic and wild-type E3 plants (Table S3). It is clear that genes involved in the alpha-amino acid metabolic process (GO: 1901605), genes that respond to endogenous stimuli (GO: 0009719) and to acidic chemicals (GO: 0001101), and genes related to the tyrosine metabolic process (GO: 0006570), calmodulin binding (GO: 0005516), lyase activity (GO: 0016829), and pyridoxal phosphate binding (GO: 0030170) were induced by flg22 in both the E3 and *Pi04089* transgenic line (Table S3).

**Fig. 5:**
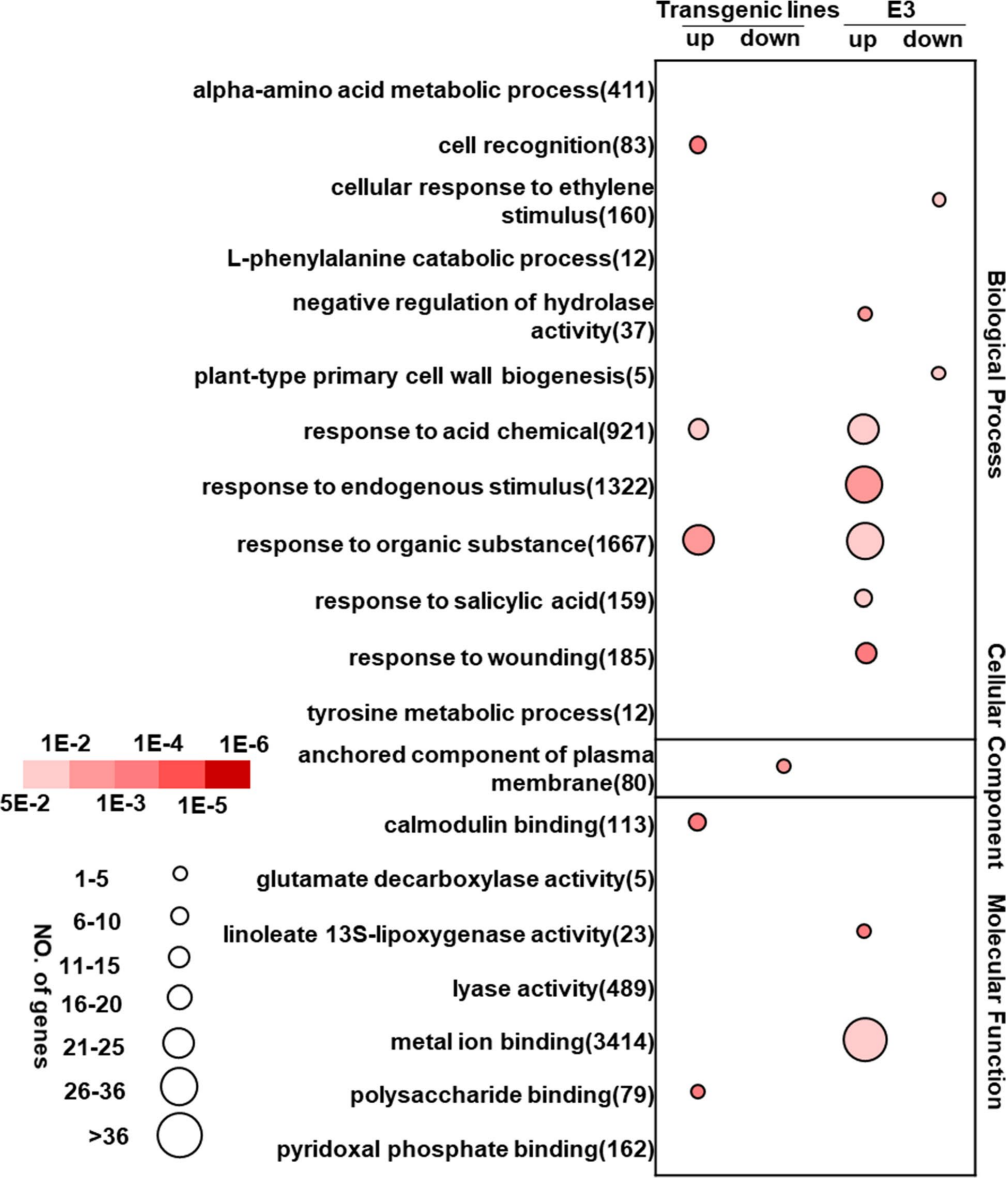
Enrichment of selected GO terms of flg22-resposive DEGs specific to *Pi04089* transgenic and wild-type E3 plants (generated with omicshare). Circle sizes indicate the number of genes within each GO term and the color of the circle indicates the adjusted *p*-values for the enrichment of the respective GO terms. See Supplementary Table 3 for the full GO terms

Enrichment of selected GO terms in flg22-resposive DEGs specific to *Pi04089* transgenic plants and the E3 line are shown in Fig. 5. Interestingly, the 369 upregulated genes only induced by flg22 in E3 were mainly responding to salicylic acid (GO: 0009751), to acidic chemicals (GO: 0071229), to endogenous stimuli (GO: 0009719), to wounding (GO: 0009611), or metal ion binding (GO: 0046872). However, genes involved in cellular responses to ethylene stimuli (GO: 0071446) and plant-type primary cell wall biogenesis (GO: 0009833) were specifically downregulated in the E3 line.

However, the 181 upregulated genes only induced by flg22 in the *Pi04089* transgenic lines were mainly related to cell recognition (GO: 0008037), polysaccharide binding (GO: 0030247), and calmodulin binding (GO: 0005516). Specific downregulated genes in transgenic plants were anchored to components of the plasma membrane (GO: 0046658). In summary, the above results indicate that *Pi04089* alters the potato plants’ ability to respond to flg22.

**Fig. 6:**
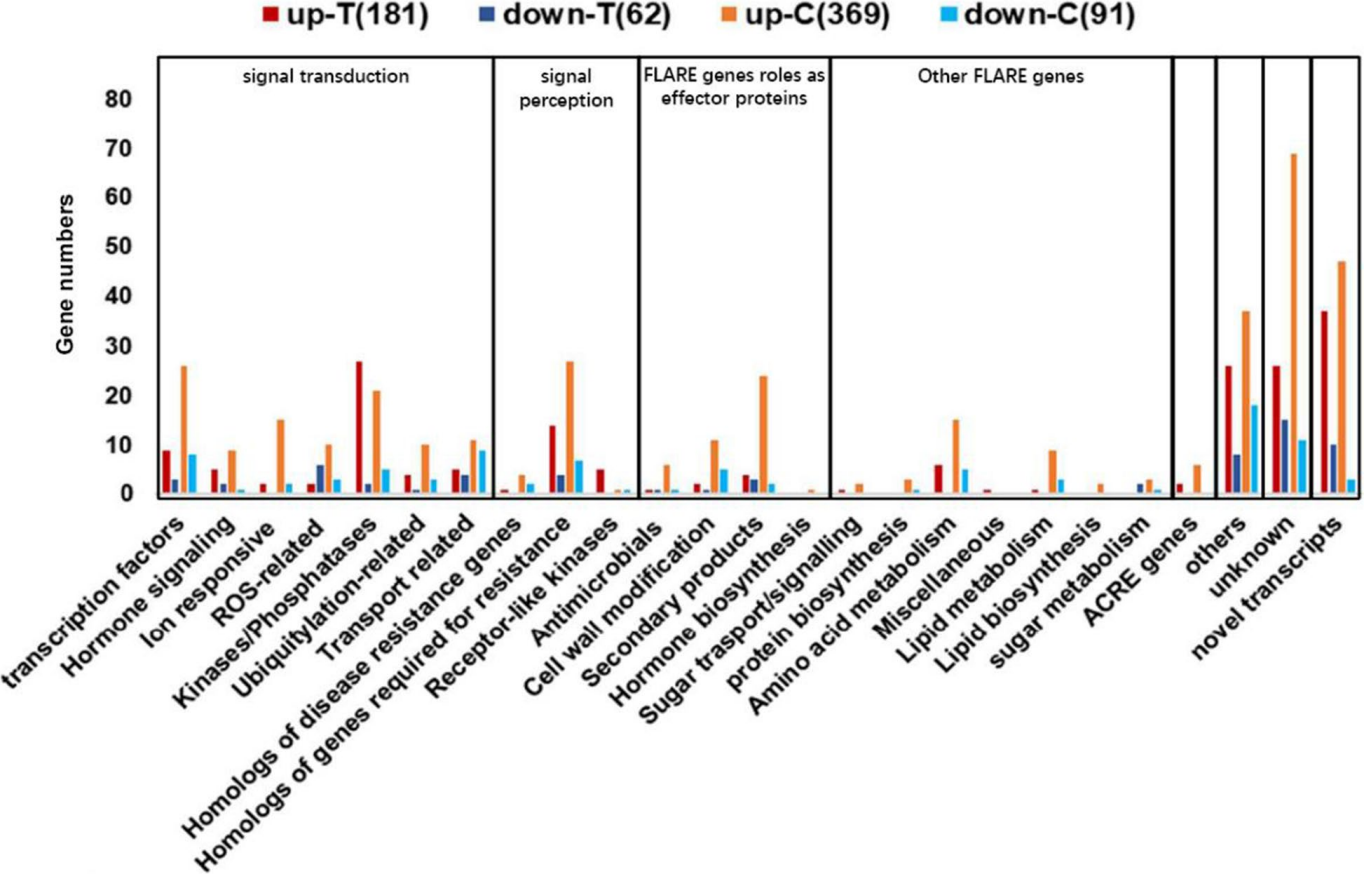
The classification of specific DEGs triggered by flg22 in *Pi04089* transgenic and wild-type E3 plants. Down-T or up-T represent the number of genes downregulated or upregulated in flg22-treated *Pi04089* stable transgenic plants. Up-C or down-C represent the number of genes downregulated or upregulated in flg22-treated E3 plants

In order to further explore the specific gene responses to flg22 in *Pi04089* transgenic and E3 plants, DEGs were divided into different classes (Fig. 6, Table S6). The results show that certain ACRE genes, transcription factor genes, ion-responsive genes, and homologs of genes required for resistance, and genes related to secondary products and lipid metabolism were upregulated in the E3 line only, which indicates that a larger number of resistance-related genes induced by flg22 in wild-type potato plant were suppressed by Pi04089. On the other hand, certain genes, for example, genes encoding Kinases/Phosphatases, were upregulated in the *Pi04089* transgenic line only, demonstrating that there are various responses that are specific to *Pi04089* transgenic plants.

Furthermore, qRT-PCR confirmed that nine defense-related genes were significantly upregulated in the E3 line as compared to the two *Pi04089* transgenic lines following the flg22 treatment (Fig. S5). These genes coded for Avr9/Cf-9 rapidly elicited protein 231 (PGSC0003DMG400001396), Avr9/Cf-9 rapidly elicited protein 75 (PGSC0003DMG400016149, PGSC0003DMG400016792), Chitinase (PGSC0003DMG400011842), Alpha-DOX2 (PGSC0003DMG400000506), ATP binding protein (PGSC0003DMG400025472), StFRK (PGSC0003DMG400001732), StWRKY17 (PGSC0003DMG400024961), and StWRKY33 (PGSC0003DMG400011633). The results confirm that the expression of *Pi04089* in potato plants inhibits the expression of many defense-related genes in response to flg22.

### Transient expression of certain DEGs in *N. benthamiana* alters the disease-resistance level

**Fig. 7:**
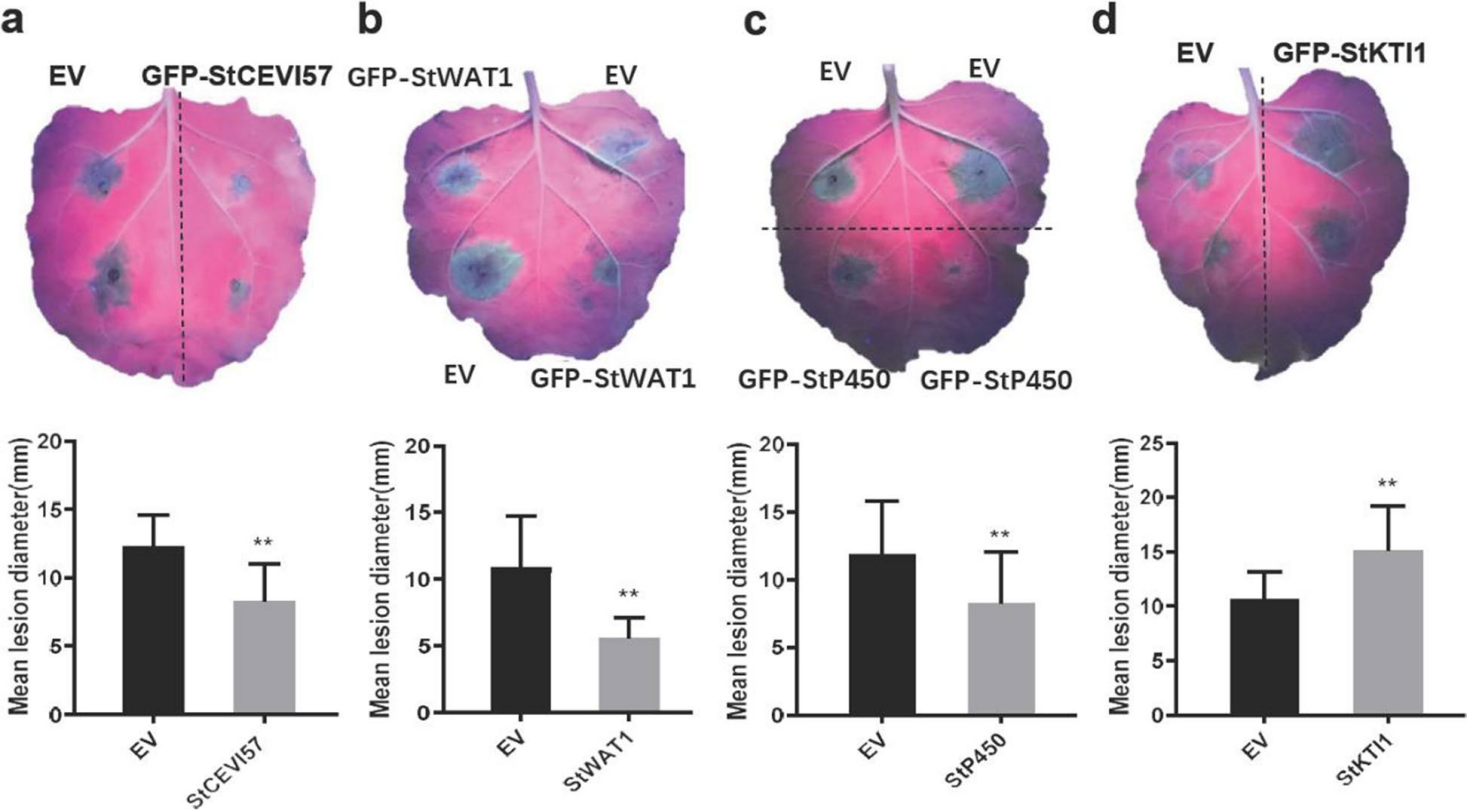
The transient expression of four DEGs affects *P. infestans* colonization in *N. benthamiana* leaves. (**a** – **d**) Images show the disease lesion on *N. benthamiana* leaves transient expressing *GFP*-*StCEVI57*, *GFP*-*StP450*, *GFP*-*StWAT1,* and *GFP-StKTI1* constructs with GFP-EV as the control. Bar graphs show disease lesion size at 5 days after *P. infestans* 88,069 inoculation. Transient expression of four genes significantly affects *P. infestans* colonization (one way ANOVA, **, *p* < 0.01). Error bars represent mean ± SD of three replicates (each replication contains 20 leaves from five plants)

Stable expression of *Pi04089* in potato plants triggers many DEGs, including ACRE genes, resistance-related genes, transcript factors genes, enzymes, and other genes (Table S2). To test whether these genes are involved in plant disease resistance, the top 15 differentially expressed genes (six up- and nine downregulated) in *Pi04089* transgenic plants were selected to test their function in *N. benthamiana* (Table S2). These genes were transiently expressed using the *Agrobacterium* infiltration method in *N. benthamiana* leaves. Leaves were inoculated with *P. infestans* 88,069 (100 spores/μl) 24 h after agro-infiltration. Lesion sizes were measured at 4–5 days after inoculation. The result show that, among the 15 tested genes, the lesion diameters of three downregulated genes (*StWAT1*, *StCEVI57*, and *StP450*) transiently expressed on the leaves were significantly decreased as compared to the empty vector (EV) control, i.e., *StWAT1* (PGSC0003DMG400028102), which codes for a Nodulin protein; the homolog of *WAT1-related* gene in *Arabidopsis*, *StCEVI57* (PGSC0003DMG400015290), which codes for proteinase inhibitor type-2 CEVI57; and *StP450* (PGSC0003DMG400020213), which codes for Cytochrome P450. The lesion diameter of the downregulated gene *StKTI1* (PGSC0003DMG400010136) transiently expressed on the leaves was increased significantly (Fig. 7). *StKTI1* codes for a Stigma expressed protein, which is the homolog of *Arabidopsis* Kunitz trypsin inhibitor 1 protein (*KTI1*). The above results demonstrate that transient expression of three Pi04089 suppressed genes, *StWAT1*, *StCEVI57,* and *StP450,* inhibit *P. infestans* colonization, while the expression of the Pi04089-activated gene *StKTI1* promotes pathogen invasion, demonstrating that certain Pi04089*-*induced DEGs play a role in late blight resistance.

## Discussion

*P. infestans* delivers toxic proteins and effectors into host cells to manipulate immunity [18]. Fifty-two *P. infestans* RXLR effectors (PiRXLRs) were confirmed as being induced during the early stages of infection in *N. benthamiana*. The majority promote pathogen colonization [17]. The effector *Pi04089* promotes pathogen colonization and interacts with a KH domain RNA binding protein*,* StKRBP1, which is regarded as a susceptibility factor [27]. The result was confirmed in stable *Pi04089* transgenic potato plants (Fig.1). However, the downstream signaling transduction is still poorly understood. The KH domain RNA binding protein is one of the RNA-binding proteins (RBPs), which is associated with alternative splicing, RNA modification, polyadenylation, mRNA export, mRNA localization, mRNA translation, and mRNA turnover [39]. Nine *P. infestans* effectors were identified as splicing regulatory effectors (SREs). Moreover, the SRE3 physically binds U1-70K to manipulate the plant alternative splice (AS) machinery and subsequently modulates AS-mediated plant immunity. *Pi04089* is one of the splicing regulatory effectors (SREs) [40]. Thus, we supposed that *Pi04089* manipulates plant immunity via its interaction with *StKRBP1,* which involves alternative splicing events in defense-related genes.

In this study, a comparative transcriptome analysis was conducted using high-throughput sequencing technology. In total, 1380 DEGs were identified in the *Pi04089* transgenic line. AS events in *Pi04089* transgenic plants were analyzed. A total of 263 AS events occurred in the *Pi04089* transgenic plants (filtered with FDR < 0.05) (Fig. S6A and B). Most genes associated with AS events are transcription factors, kinases/phosphatases, transport-related genes, and secondary products. However, these genes are not well characterized in terms of plant disease resistance (Table S7, Fig. S6C). Moreover, the expression of the transcriptions related to plant immunity are obviously altered in *Pi04089* transgenic plants. The manipulation of the expression of resistance-related genes by the effector *Pi04089* may have predominated AS events to manipulate host immunity in this study. However, it is worth investigating whether AS events are involved in plant immunity.

Effectors have been shown to manipulate plant immunity by directly targeting host DNA to regulate gene expression. Rust fungal effector Mlp124478 binds plant DNA and modulates transcription [41]. Oomycete CRN effector PsCRN108 targets the promoter of plant *HSP* genes to reprogram their expression [42]. *Arabidopsis* RNA binding protein can regulate the expression of ASCORBATE PEROXIDASE2, GLUTATHIONE S-TRANSFERASE TAU9, and several SMALL AUXIN UPREGULATED RNA-like genes [43]. Thus, we supposed that Pi04089 modulates gene expression with the RNA binding protein StKRBP1 through unknown mechanisms, possibly through the effects of mRNA stability or mRNA turnover.

Different effectors may influence the expression of diverse host genes to manipulate different immunity pathways. The stable expression of AVR2 in potato suppresses immunity through the overactive BR signaling pathways [29]. PITG_15718.2 suppresses many genes that positively regulate immunity and plant growth, and actives a number of genes that negatively regulate host immunity or vegetative growth by decreasing the Indole-3-Acetic acid content [30]. In our study, the Go enrichment analysis indicated that DEGs in *Pi04089* transgenic potato plants were focused around 30 GO terms (Fig. 2. Table S1). Genes involved in the glutamate metabolic process, protein disulfide reductase activity, the lipid metabolic process, the carboxylic acid metabolic process, the fatty acid metabolic process, and genes that respond to acidic chemicals, endogenous stimuli, and hormones were suppressed. Many gene functions are clearly related to plant immunity, which may help to explain the virulence function of *Pi04089*. Moreover, many genes related to the photosystem were upregulated in *Pi04089* transgenic lines (Fig. 3). This, may reflect the plant’s common response to pathogen effectors.

Salicylic acid (SA) plays an important role in plant immunity. Cell death induced by SA and phytoalexin induced by ethylene were necessary for the response of *N. benthamiana* against *P. infestans* [44]. In this study, nine genes responses to salicylic acid (GO: 0009751) were suppressed in the *Pi04089* stable transgenic line (Table S3), with their expression patterns being confirmed by qRT-PCR (Fig. 4a), including the well-known SA signaling pathway marker gene *PR1* (Fig. 4b). Furthermore, a number of transcription factors response to SA were inhibited in the transgenic lines, especially WRKY transcription factors, such as *StWRKY72*, *StWRKY27*, and *StWRKY IIe-1* (Table S2). The results indicate that *Pi04089* partially suppresses plant immunity via manipulating the expression of SA-responsive genes.

A synthetic 22-amino-acid peptide (flg22) from the highly conserved flagellin domain triggered a significant plant immunity response. Most genes rapidly responded to flg22 (FLARE genes)-encoded transcription factors and protein kinases/phosphatases [31]. Moreover, FLS2 interacts with flg22 to increase the production of reactive oxygen species (ROS) and the expression of mitogen-activated protein (MAP) kinases [45]. Many PTI genes triggered by flg22 are conserved in different species. Other genes, such as transcription factors, secondary metabolites, and certain enzymes, may be responsible for the emergence of species-specific gene induction by flg22 [46]. As in other plant species, flg22 triggered strong defense responses in wild-type potato plants, which was characterized by the activation of many flg22-induced marker genes (Table S4). Extracellular Ca^2+^ has be shown to significantly contribute to plant immunity as triggered by plant elicitor peptide (Pep3), more so than flg22, which indicates that extracellular Ca^2+^ influx plays an important role in flg22 activation of plant cell defenses [47]. We found that over 10 calmodulin-related protein genes and many ROS-related genes were upregulated in flg22-treated potato plants (Table S4).

When we compared the flg22-responsive differences between the *Pi04089* transgenic potato plants and the wild-type E3 control plants, we found that they shared many up- and downregulated genes, including many defense-related genes, such as signal transduction and ACRE genes (Fig. 6, Table S5, Table S6). However, many defense-related genes that responded to flg22 in the E3 control line were suppressed in the *Pi04089* transgenic lines (Fig. 6, Table S6). Various ACRE genes (*ACRE 75/137/180/189/231*) were only triggered in the E3 line by flg22. These results demonstrate that the expression of *Pi04089* in potato plants alters the responsive ability of potato to flg22. Many pathogen effectors could suppress flg22-triggered PTI genes. Chen et al. found that the *Globodera rostochiensis* effector GrCEP12 suppresses flg22-triggered marker gene (*NbPti5* and *NbAcre31*) expression in *N. benthamiana* [48]. Zheng et al. reported that eight *P. infestans* RXLR effectors suppress early Flg22-induced immune response. This was demonstrated by suppressing the flg22-dependent activation of the *Luc* reporter gene under control of a PAMP-inducible promoter *pFRK1* in tomato and Arabidopsis protoplasts; contrarily, PITG_04089 (Pi04089) did not induce the same effects [49]. In this study, we found that Pi04089 could suppress the expression of many flg22-inducible genes, such as ACRE genes, and *StFRK*, *StWRKY17,* and *StWRKY33*. On the contrary, those genes were activated in wild-type potato plants upon flg22 treatment (Fig.5, Fig.6, Table S6). The difference likely results from the different experimental systems used. A protoplast transient expression system was used to test luciferase activity upon flg22 treatment by Zheng et al. [49]. Instead, stable effector transgenic lines were used in present study. Another explanation is that, may be, Pi04089 represses genes’ expression upstream of *StFRK* upon flg22 treatment. In present study, the RNAseq method provides comprehensive information and the possibility to uncover more flg22-inducible genes that were suppressed in the *Pi04089* transgenic potato plants.

In the present study, 15 DGEs in *Pi04089* transgenic potato plants were selected in order to test their defensive function against *P. infestans*. Functional verification showed that the transient expression of three downregulated genes (*StWAT1*, *StCEVI57,* and *StP450*) in *Pi04089* transgenic potato plants contributes to late blight resistance in *N. benthamiana.* This was demonstrated by suppressing *P. infestans* 88,069 colonization (Fig. 7). Walls Are Thin1 (*WAT1*), a major gene required for cell wall deposition, showed broad-spectrum resistance against vascular pathogens in Arabidopsis [50]. However, another study showed that NtKTI1 was a positive factor against *Rhizoctonia solani* [51]. Cytochrome P450s (CYPs) are involved in the oxidation-reduction process and play important roles in abiotic and biotic stress responses by catalyzing NADPH- or O_2_-dependent hydroxylation reactions, which could be induced by methyl jasmonate or fungal infections [52]. The expression of *GmCYP82A3* enhanced resistance against black shank (*Phytophthora parasitica*) and gray mold (*Botrytis cinereal*) [53], and they are also involved in epicuticular wax biosynthesis, hypersensitive rapid cell death, and the wounding process [54–56]. *StCEVI57* codes for a predicted proteinase inhibitor type-2 CEVI57. Tomato *SlCEVI57* has been reported to be ectopically expressed in tomato aerial tissues upon viroid infection and ethephon treatment [57]. Moreover, the transient expression of the upregulated gene *StKTI1* decreases late blight resistance (Fig. 7). KTI1 is a serine protease (Kunitz trypsin) inhibitor. SA treatment caused the robust expression of *AtKTI1* at 24 h in Arabidopsis. The overexpression of *AtKTI1* enhances the susceptibility of Arabidopsis to *Ecc*, while RNAi silencing causes enhanced disease resistance against *Ecc* SCC1 [58]. Although not all of the selected DEGs were directly involved in late blight resistance, the present results confirmed that Pi04089 suppresses host resistance, at least in part, by altering the expression of many defense-related genes.

In summary, this research provides new insights into how an oomycete effector manipulates host immunity. *Pi04089* subverts host immunity by suppressing the expression of many defense-related genes (such as *StP450, StCEVI57,* and *StWAT1*) and activating susceptible gene (such as *StKTI1*) to facilitate pathogen invasion. *Pi04089* also suppresses plant PTI responses to PAMPs. Future studies will focus on the molecular mechanisms involved in how *Pi04089* interacts with *StKRBP1* to regulate gene expression and suppress host immunity.

## Conclusion

In this study, we demonstrated that potato plants stably expressing *Pi04089* were more sensitive to *P. infestans* than their wild-type counterparts. A total of 658 upregulated genes and 722 downregulated genes were identified in *Pi04089* transgenic plants. Many resistance-related genes were suppressed and the genes involved in the SA pathway were also inhibited in *Pi04089* transgenic plants. Flg22 induces a great deal of defense-related genes in potato plants, including many well-known PTI genes, gene responses to biotic/abiotic stress, and important genes in secondary metabolite synthesis/metabolism in the E3 line. Pi04089 suppresses flg22-triggered PTI responses by inhibiting the expression of a number of defense-related genes, including ACRE genes. Three downregulated and one upregulated genes in *Pi04089* transgenic plants were confirmed to involve in *P. infestans* resistance in *N. benthamiana*. Our finding provides new insights into how an oomycete effector perturbs host immune responses at the transcriptome level.

## Supplementary Information

**Additional file 1: Fig. S1**. Expression of *Pi04089* in three potato (E3) transgenic lines. **Fig. S2**. Validating the expression of selected DEGs in three transgenic lines by qRT-PCR. **Fig. S3**. Expression level of eight gene responses to flg22 treatment in potato E3. **Fig. S4**. The Venn of DEGs induced by flg22 in *Pi04089* transgenic and control E3 plant. **Fig. S5**. Nine defense-related genes were specifically upregulated in flg22-treated E3 plants but not in the *Pi04089* transgenic lines. **Fig. S6**. Alternative splicing events occur in *Pi04089* transgenic plants.

**Additional file 2: Table S1** RNA sequence map information and expression profile.

**Additional file 3: Table S2** DEGs in the *Pi04089* transgenic line.

**Additional file 4: Table S3** GO analysis of DGEs.

**Additional file 5: Table S4** DEGs triggered by flg22 in the E3 line.

**Additional file 6: Table S5** DEGs triggered by flg22 in the *Pi04089* transgenic line.

**Additional file 7: Table S6** Classification of common and specific genes in the flg22-treated *Pi04089* transgenic line and control plants.

**Additional file 8: Table S7** Alternative splicing events.

**Additional file 9: Table S8** Primers used in this study.

## Data Availability

The datasets supporting the conclusions of this article are included within the article (and its supplemental files). The Illumina sequence data generated during the current study are accessible through BioProject accession number PRJNA754031 (http://www.ncbi.nlm.nih.gov/).
