# Supplementary material for: Phytophthora infestans RXLR effector Pi04089 perturbs diverse defense-related genes to suppress host immunity
Source: BMC Plant Biol. 2021 Dec 9;21:582. doi: 10.1186/s12870-021-03364-0 (PMC8656059; doi:10.1186/s12870-021-03364-0)
Supplement: Supplementary file 1 — Additional file 1: Fig. S1. Expression of Pi04089 in three potato (E3) transgenic lines. Fig. S2. Validating the expression of selected DEGs in three transgenic lines by qRT-PCR. Fig. S3. Expression level of eight gene responses to flg22 treatment in potato E3. Fig. S4. The Venn of DEGs induced by flg22 in Pi04089 transgenic and control E3 plant. Fig. S5. Nine defense-related genes were specifically upregulated in flg22-treated E3 plants but not in the Pi04089 transgenic lines. Fig. S6. Alternative splicing events occur in Pi04089 transgenic plants. [file 12870_2021_3364_MOESM1_ESM.pdf]

Fig. S1

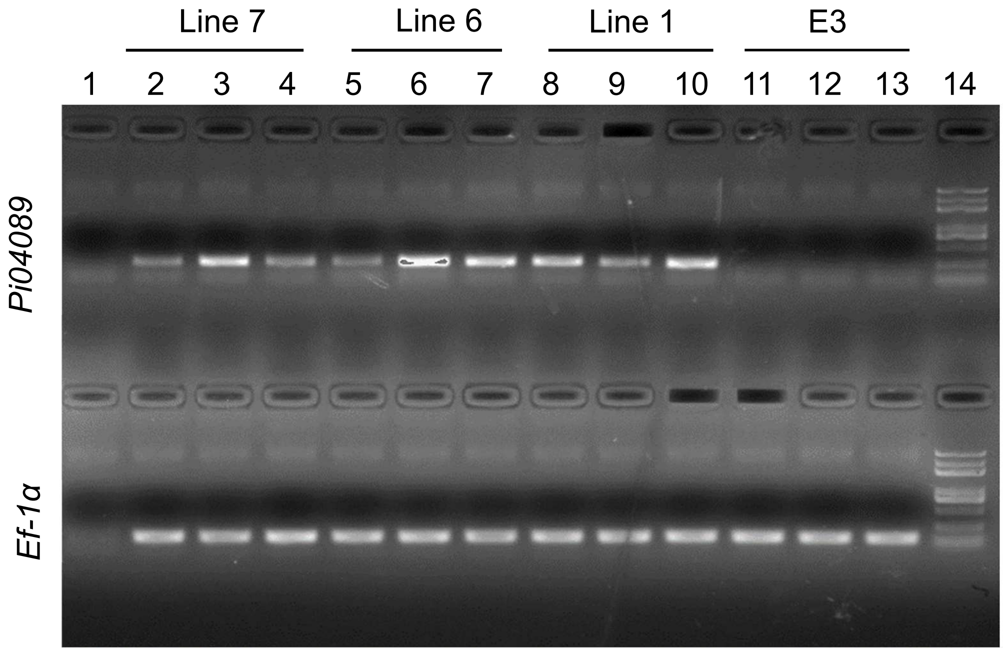

Fig. S1 Expression of *Pi04089* in three transgenic potato lines. Semi-quantitative RT-PCR was used to detect expression of *Pi04089* in 3 transgenic lines using *Pi04089*-specific primers (full CDS). The *StEF-1α* gene was used as endogenous control. Three biological repeats for each line. Lane 1. Negative control, lane 2/3/4. Pi04089-line7, lane 5/6/7. Pi04089-line6, lane 8/9/10. Pi04089-line1, lane 11/12/13. E3. Lane 14. DNA maker.

The original image for Fig. S1

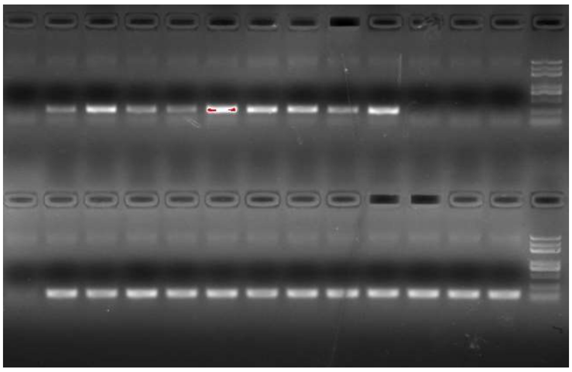

Fig. S2

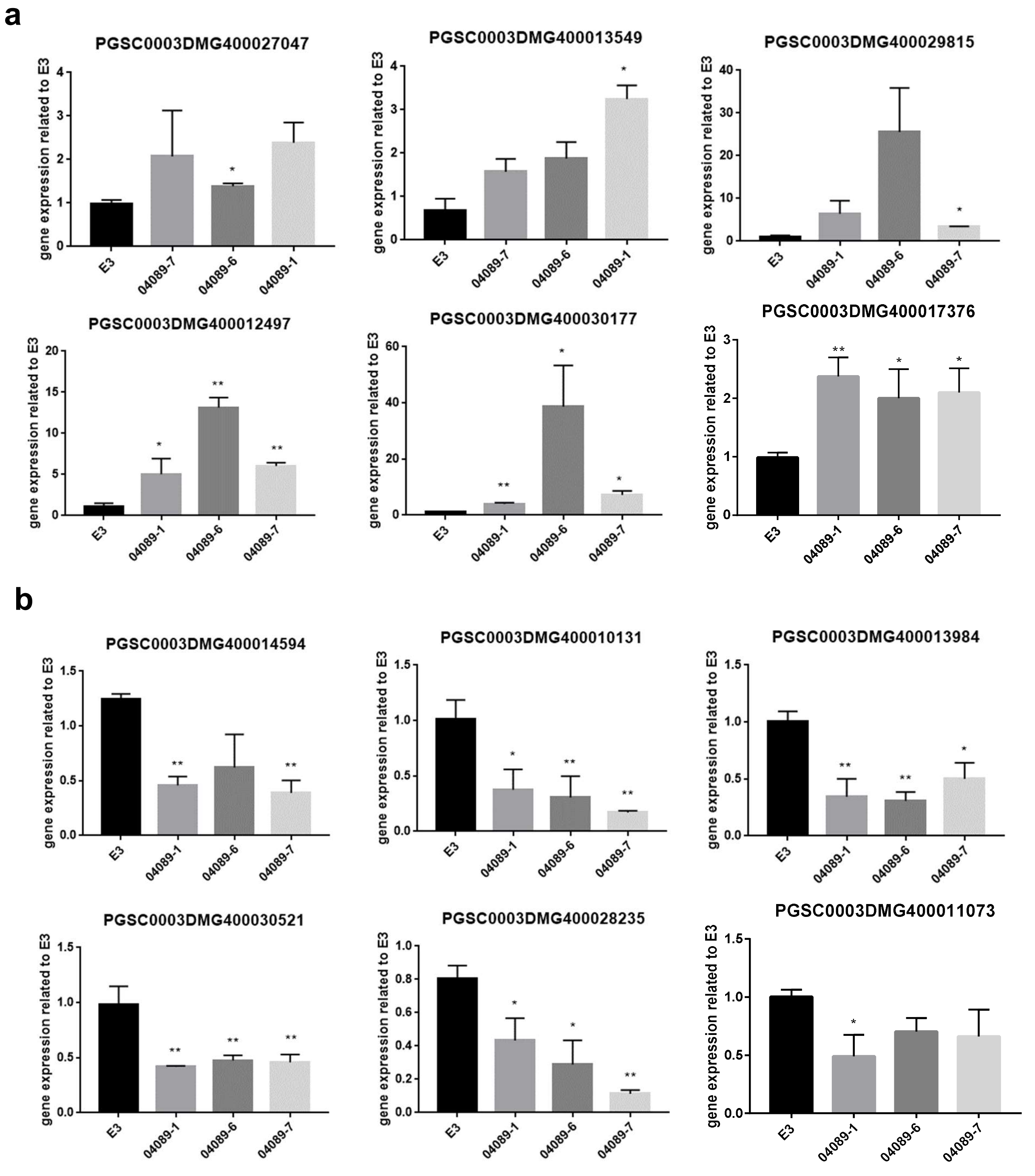

Fig. S2 Validating the expression of selected DEGs in three transgenic lines by qRT-PCR. (**a**) Six up regulated genes. (**b**) Six down regulated genes. * indicates significant (one-way ANOVA, $p < 0.05$) differences, ** indicates extremely significant (one-way ANOVA, $p < 0.01$) differences. Error bars represent SE of the mean of three biological repeats.

Fig. S3

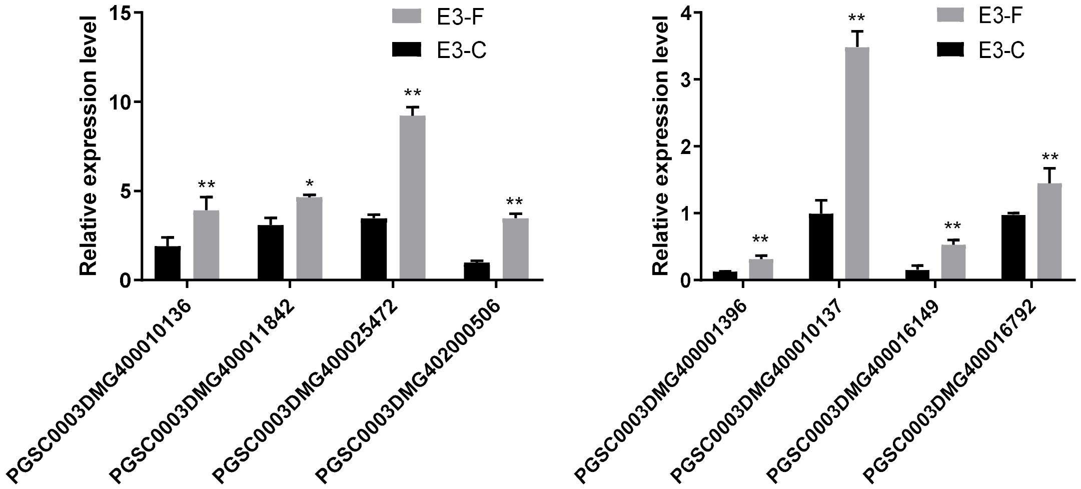

Fig. S3 Expression of eight genes response to flg22 treatment in potato E3. Eight up-regulated defense-related genes were selected (*StEF-1a* used as the reference gene) for qRT-PCR. Samples were collected 30 min after flg22 treatment. * indicates significant differences (one-way ANOVA, $p < 0.05$), ** indicates extremely significant differences (one-way ANOVA, $p < 0.01$). Error bars represent mean ± SE of three biological repeats.

Fig. S4

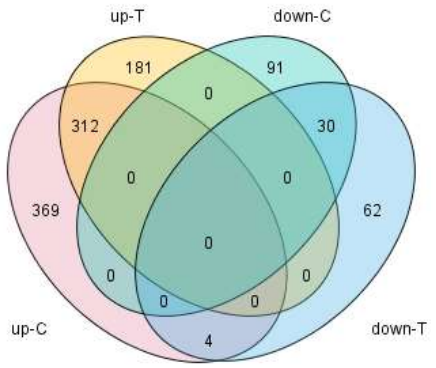

Fig. S4 The Venn of DEGs induced by flg22 in *Pi04089* transgenic and E3 control plants. Down-T or up-T represents the number of down regulated or up regulated genes in the flg22 treated *Pi04089* stable transgenic potato plants. Up-C and down-C represents the number of down regulated or up regulated genes in flg22 treated control E3 plants.

Fig. S5

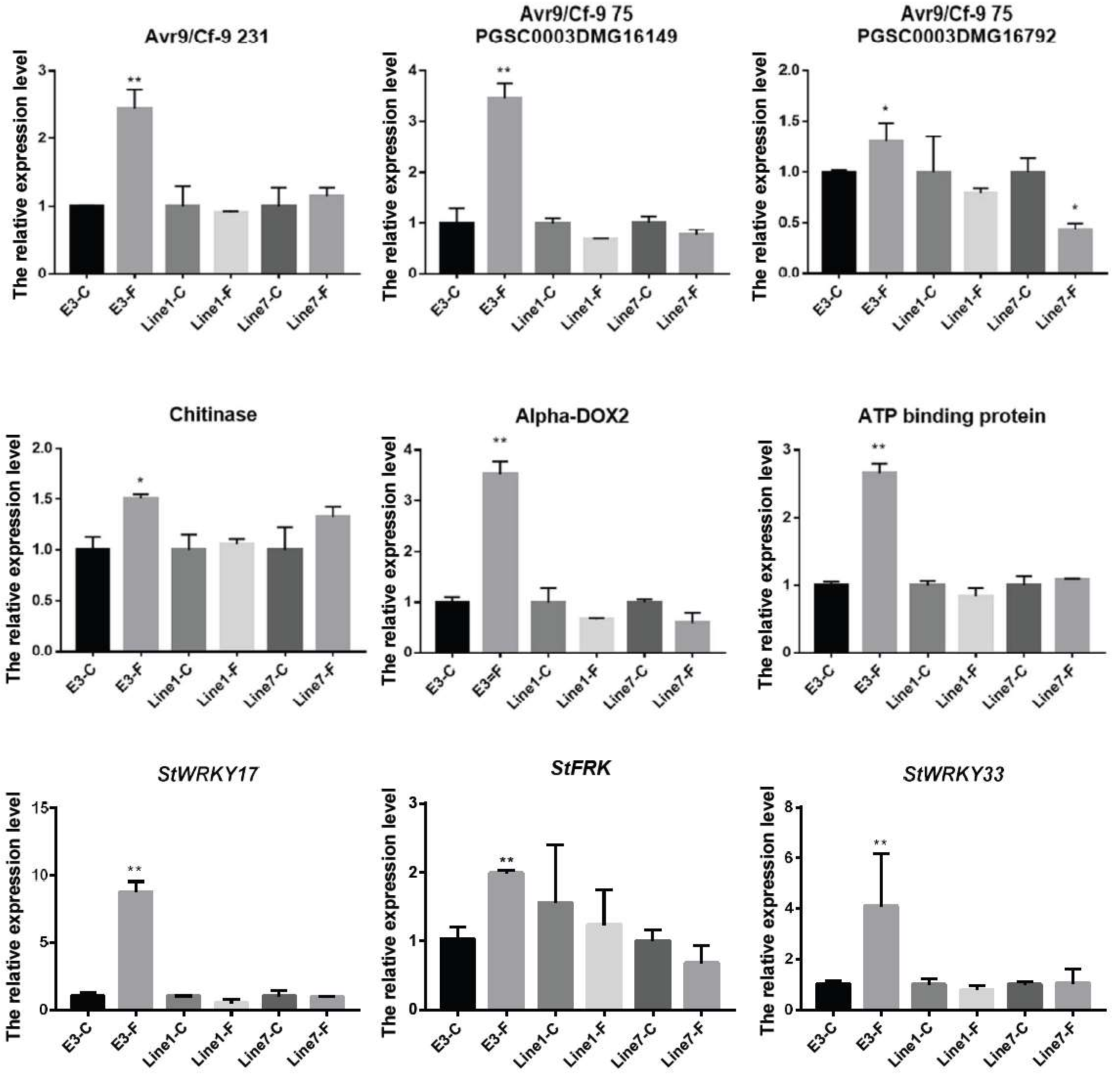

Fig. S5 Nine defense-related genes were significantly up-regulated in flg22 treated E3 plants but no in the *Pi04089* transgenic lines. The relative expression level of each gene responded to flg22 treatment was compared to that of control E3 or transgenic plant without flg22 treatment. Expression level was normalized to *StEF-1α*. Leaves from E3 or transgenic plant were treated with 50 mM flg22 for 30 min, and then samples were collected for RNA extraction and qRT-PCR. Water sprayed samples were used as control (C). (t-test, ** p < 0.01, * p < 0.05). The data represent mean ± SE from three biological repeats.

Fig. S6

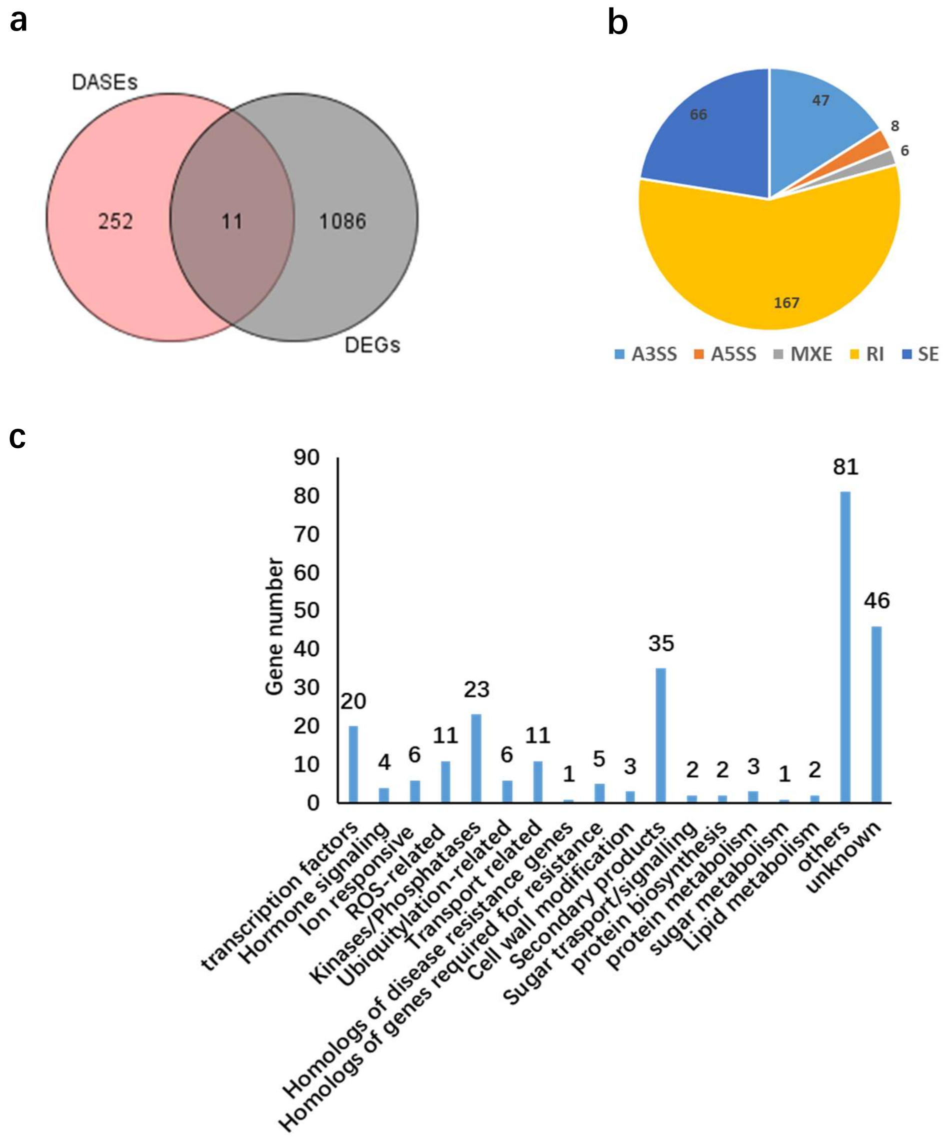

Fig. S6 Alternative splicing events occur in *Pi04089* transgenic plants. (**a**) Venn diagram showing differentially expressed genes (DEGs) and differentially alternatively spliced genes (DASGs) in the *Pi04089* transgenic plants. DASG IDs was provided in Supplemental Table 8. (**b**) Different alternative splicing events in the *Pi04089* transgenic plant. A3SS: Alternative 3' splice site. A5SS: Alternative 5' splice site. MXE: Mutually exclusive exons. RI: Retained intron. SE: Skipped exon. (**c**) The classification of DASGs. Most of them are transcription factors, Kinases/phosphatases, secondary productions, ROS- and transport-related genes.
